# The Manila Declaration on the Drug Problem in the Philippines

**DOI:** 10.5334/aogh.28

**Published:** 2019-03-05

**Authors:** Nymia Simbulan, Leonardo Estacio, Carissa Dioquino-Maligaso, Teodoro Herbosa, Mellissa Withers

**Affiliations:** 1 University of Southern California, US; 2 University of the Philippines, PH

## Abstract

When Philippine President Rodrigo R. Duterte assumed office in 2016, his government launched an unprecedented campaign against illegal drugs. The drug problem in the Philippines has primarily been viewed as an issue of law enforcement and criminality, and the government has focused on implementing a policy of criminalization and punishment. The escalation of human rights violations has caught the attention of groups in the Philippines as well as the international community. The Global Health Program of the Association of Pacific Rim Universities (APRU), a non-profit network of 50 universities in the Pacific Rim, held its 2017 annual conference in Manila. A special half-day workshop was held on illicit drug abuse in the Philippines which convened 167 participants from 10 economies and 21 disciplines. The goal of the workshop was to collaboratively develop a policy statement describing the best way to address the drug problem in the Philippines, taking into consideration a public health and human rights approach to the issue. The policy statement is presented here.

## Background

When Philippine President Rodrigo R. Duterte assumed office on June 30, 2016, his government launched an unprecedented campaign against illegal drugs. He promised to solve the illegal drug problem in the country, which, according to him, was wreaking havoc on the lives of many Filipino families and destroying the future of the Filipino youth. He declared a “war on drugs” targeting users, peddlers, producers and suppliers, and called for the Philippine criminal justice system to put an end to the drug menace [1].

According to the Dangerous Drugs Board (DDB) (the government agency mandated to formulate policies on illegal drugs in the Philippines), there are 1.8 million current drug users in the Philippines, and 4.8 million Filipinos report having used illegal drugs at least once in their lives [2]. More than three-quarters of drug users are adults (91%), males (87%), and have reached high school (80%). More than two-thirds (67%) are employed [2]. The most commonly used drug in the Philippines is a variant of methamphetamine called *shabu* or “poor man’s cocaine.” According to a 2012 United Nations report, the Philippines had the highest rate of methamphetamine abuse among countries in East Asia; about 2.2% of Filipinos between the ages 16–64 years were methamphetamines users.

The drug problem in the Philippines has primarily been viewed as an issue of law enforcement and criminality, and the government has focused on implementing a policy of criminalization and punishment. This is evidenced by the fact that since the start of the “war on drugs,” the Duterte government has utilized punitive measures and has mobilized the Philippine National Police (PNP) and local government units nationwide. With orders from the President, law enforcement agents have engaged in extensive door-to-door operations. One such operation in Manila in August 2017 aimed to “shock and awe” drug dealers and resulted in the killing of 32 people by police in one night [3].

On the basis of mere suspicion of drug use and/or drug dealing, and criminal record, police forces have arrested, detained, and even killed men, women and children in the course of these operations. Male urban poor residents in Metro Manila and other key cities of the country have been especially targeted [4]. During the first six months of the Duterte Presidency (July 2016–January 2017), the PNP conducted 43,593 operations that covered 5.6 million houses, resulting in the arrest of 53,025 “drug personalities,” and a reported 1,189,462 persons “surrendering” to authorities, including 79,349 drug dealers and 1,110,113 drug users [5]. Government figures show that during the first six months of Duterte’s presidency, more than 7,000 individuals accused of drug dealing or drug use were killed in the Philippines, both from legitimate police and vigilante-style operations. Almost 2,555, or a little over a third of people suspected to be involved in drugs, have been killed in gun battles with police in anti-drug operations [5,6]. Community activists estimate that the death toll has now reached 13,000 [7]. The killings by police are widely believed to be staged in order to qualify for the cash rewards offered to policeman for killing suspected drug dealers. Apart from the killings, the recorded number of “surrenderees” resulting in mass incarceration has overwhelmed the Philippine penal system, which does not have sufficient facilities to cope with the population upsurge. Consequently, detainees have to stay in overcrowded, unhygienic conditions unfit for humans [8].

The escalation of human rights violations, particularly the increase in killings, both state-perpetrated and vigilante-style, has caught the attention of various groups and sectors in society including the international community. Both police officers and community members have reported fear of being targeted if they fail to support the state-sanctioned killings [9]. After widespread protests by human rights groups, Duterte called for police to shoot human rights activists who are “obstructing justice.” Human Rights organizations, such as Human Rights Watch and Amnesty International, have said that Duterte’s instigation of unlawful police violence and the incitement of vigilante killings may amount to crimes against humanity, violating international law [10,11]. The European Union found that human rights have deteriorated significantly since Duterte assumed power, saying “The Philippine government needs to ensure that the fight against drug crimes is conducted within the law, including the right to due process and safeguarding of the basic human rights of citizens of the Philippines, including the right to life, and that it respects the proportionality principle [12].” Despite the fact that, in October 2017, Duterte ordered the police to end all operations in the war on drugs, doubts remain as to whether the state-sanctioned killings will stop [13]. Duterte assigned the Philippine Drug Enforcement Agency (PDEA) to be the sole anti-drug enforcement agency.

Duterte’s war on drugs is morally and legally unjustifiable and has created large-scale human rights violations; and is also counterproductive in addressing the drug problem. International human rights groups and even the United Nations have acknowledged that the country’s drug problem cannot be resolved using a punitive approach, and the imposition of criminal sanctions and that drug users should not be viewed and treated as criminals [14]. Those critical of the government’s policy towards the illegal drug problem have emphasized that the drug issue should be viewed as a public health problem using a rights-based approach (RBA). This was affirmed by UN Secretary General Ban Ki Moon on the 2015 International Day Against Drug Abuse and Illegal Trafficking when he stated, “…We should increase the focus on public health, prevention, treatment and care, as well as on economic, social and cultural strategies [15].” The United Nations Human Rights Council released a joint statement in September 2017, which states that the human rights situation in the Philippines continued to cause serious concern. The Council urged the government of the Philippines to “take all necessary measures to bring these killings to an end and cooperate with the international community to pursue appropriate investigations into these incidents, in keeping with the universal principles of democratic accountability and the rule of law [16].” In October 2017, the Philippines Dangerous Drug Board (DDB) released a new proposal for an anti-drug approach that protects the life of the people. The declaration includes an implicit recognition of the public health aspect of illegal drug use, “which recognizes that the drug problem as both social and psychological [16].”

## Workshop on Illicit Drug Abuse in the Philippines

The Association of Pacific Rim Universities (APRU) is a non-profit network of 50 leading research universities in the Pacific Rim region, representing 16 economies, 120,000 faculty members and approximately two million students. Launched in 2007, the APRU Global Health Program (GHP) includes approximately 1,000 faculty, students, and researchers who are actively engaged in global health work. The main objective of the GHP is to advance global health research, education and training in the Pacific Rim, as APRU member institutions respond to global and regional health challenges. Each year, about 300 APRU GHP members gather at the annual global health conference, which is hosted by a rotating member university. In 2017, the University of the Philippines in Manila hosted the conference and included a special half-day workshop on illicit drug abuse in the Philippines.

Held on the first day of the annual APRU GHP conference, the workshop convened 167 university professors, students, university administrators, government officials, and employees of non-governmental organizations (NGO), from 21 disciplines, including anthropology, Asian studies, communication, dentistry, development, education, environmental health, ethics, international relations, law, library and information science, medicine, nutrition, nursing, occupational health, pharmaceutical science, physical therapy, political science, psychology, public health, and women’s studies. The participants came from 10 economies: Australia, China, Hong Kong, Indonesia, Japan, Mexico, Nepal, the Philippines, Thailand, and the US. The special workshop was intended to provide a venue for health professionals and workers, academics, researchers, students, health rights advocates, and policy makers to: 1) give an overview on the character and state of the drug problem in the Philippines, including the social and public health implications of the problem and the approaches being used by the government in the Philippines; 2) learn from the experiences of other countries in the handling of the drug and substance abuse problem; and 3) identify appropriate methods and strategies, and the role of the health sector in addressing the problem in the country. The overall goal of the workshop was to collaboratively develop a policy statement describing the best way to address this problem in a matnner that could be disseminated to all the participants and key policymakers both in the Philippines, as well as globally.

The workshop included presentations from three speakers and was moderated by Dr. Carissa Paz Dioquino-Maligaso, head of the National Poison Management and Control Center in the Philippines. The first speaker was Dr. Benjamin P. Reyes, Undersecretary of the Philippine Dangerous Drugs Board, who spoke about “the State of the Philippine Drug and Substance Abuse Problem in the Philippines.” The second speaker was Dr. Joselito Pascual, a medical specialist from the Department of Psychiatry and Behavioral Medicine, at the University of the Philippines General Hospital in Manila. His talk was titled “Psychotropic Drugs and Mental Health.” The final speaker was Patrick Loius B. Angeles, a Policy and Research Officer of the NoBox Transitions Foundation, whose talk was titled “Approaches to Addressing the Drug and Substance Abuse Problem: Learning from the Experiences of Other Countries.” Based on the presentations, a draft of the Manila Declaration on the Drug Problem in the Philippines was drafted by the co-authors of this paper. The statement was then sent to the workshop participants for review and comments. The comments were reviewed and incorporated into the final version, which is presented below.

## Declaration

**“Manila Statement on the Drug Problem in the Philippines”**

Gathering in this workshop with a common issue and concern – the drug problem in the Philippines and its consequences and how it can be addressed and solved in the best way possible;

Recognizing that **the drug problem in the Philippines is a complex and multi-faceted problem that includes not only criminal justice issues but also public health issues** and with various approaches that can be used in order to solve such;

We call for **drug control policies and strategies that incorporate evidence-based, socially acceptable, cost-effective, and rights-based approaches** that are designed to minimize, if not to eliminate, the adverse health, psychological, social, economic and criminal justice consequences of drug abuse towards the goal of attaining a society that is free from crime and drug and substance abuse;

Recognizing, further, that **drug dependency and co-dependency, as consequences of drug abuse, are mental and behavioral health problems, and that in some areas in the Philippines injecting drug use comorbidities such as the spread of HIV and AIDS** are also apparent, and that current prevention and treatment interventions are not quite adequate to prevent mental disorders, HIV/AIDS and other co-morbid diseases among people who use drugs;

Affirming that the **primacy of the sanctity/value of human life and** the value of human dignity, **social protection of the victims of drug abuse and illegal drugs trade** must be our primary concern;

And that all health, psycho-social, socio-economic and rights-related interventions leading to the reduction or elimination of the adverse health, economic and social consequences of drug abuse and other related co-morbidities such as HIV/AIDS should be considered in all plans and actions toward the control, prevention and treatment of drug and substance abuse;

As a community of health professionals, experts, academics, researchers, students and health advocates, **we call on the Philippine government to address the root causes of the illegal drug problem in the Philippines utilizing the aforementioned affirmations**. We assert that the drug problem in the country is but a symptom of deeper structural ills rooted in social inequality and injustice, lack of economic and social opportunities, and powerlessness among the Filipino people. Genuine solutions to the drug problem will only be realized with the fulfillment and enjoyment of human rights, allowing them to live in dignity deserving of human beings. As members of educational, scientific and health institutions of the country, being rich and valuable sources of human, material and technological resources, we affirm our commitment to contribute to solving this social ill that the Philippine government has considered to be a major obstacle in the attainment of national development.

## Conclusion

The statement of insights and affirmations on the drug problem in the Philippines is a declaration that is readily applicable to other countries in Asia where approaches to the problem of drug abuse are largely harsh, violent and punitive.

As a community of scholars, health professionals, academics, and researchers, we reiterate our conviction that the drug problem in the Philippines is multi-dimensional in character and deeply rooted in the structural causes of poverty, inequality and powerlessness of the Filipino people. Contrary to the government’s position of treating the issues as a problem of criminality and lawlessness, the drug problem must be addressed using a holistic and rights-based approach, requiring the mobilization and involvement of all stakeholders. This is the message and the challenge which we, as members of the Association of Pacific Rim Universities, want to relay to the leaders, policymakers, healthcare professionals, and human rights advocates in the region; we must all work together to protect and promote health and well being of all populations in our region.
